# Methicillin and inducible clindamycin resistance in Gram-positive cocci among GeneXpert-positive pulmonary tuberculosis patients and apparently healthy individuals in Northwest Ethiopia

**DOI:** 10.1186/s12866-026-05071-y

**Published:** 2026-04-18

**Authors:** Tebelay Dilnessa, Feleke Moges, Belay Tessema, Workagegnehu Hailu, Baye Gelaw

**Affiliations:** 1 Department of Medical Microbiology, School of Biomedical and Laboratory Sciences, College of Medicine and Health Sciences, University of Gondar, P. O. Box 196, Gondar, Ethiopia; 2 Department of Medical Laboratory Science, College of Medicine and Health Sciences, Debre Markos University, P. O. Box 269, Debre Markos, Ethiopia; 3 Institute of Clinical Immunology, Faculty of Medicine, University of Leipzig, Leipzig, 04103 Germany; 4 Department of Internal Medicine, School of Medicine, College of Medicine and Health Sciences, University of Gondar, P. O. Box 196, Gondar, Ethiopia

**Keywords:** Gram-positive cocci, Methicillin resistance, Inducible clindamycin resistance, Multi-drug resistance, Pulmonary tuberculosis

## Abstract

**Background:**

Pulmonary tuberculosis (PTB) remains a major cause of morbidity and mortality globally. Gram-positive cocci (GPC) among tuberculosis patients may complicate clinical outcomes and contribute to antimicrobial resistance. Methicillin resistance and inducible clindamycin resistance mediated by macrolide-lincosamide-streptogramin B (MLSB) phenotypes pose significant therapeutic challenges by limiting effective treatment options and contributing to treatment failure. In Ethiopia, data on methicillin resistance and inducible clindamycin resistance in Gram-positive cocci among PTB patients and apparently healthy individuals are scarce. This study aimed to determine the prevalence, methicillin and inducible clindamycin resistance and associated factors of Gram-positive cocci among PTB patients and apparently healthy individuals in Northwest Ethiopia.

**Methods:**

A cross-sectional study was conducted among 300 participants, comprising 150 GeneXpert-positive PTB patients and 150 apparently healthy individuals, recruited from comprehensive specialized hospitals and the community, respectively. Sputum specimens were collected aseptically and cultured on blood and chocolate agar plates. Isolates were identified using standard microbiological methods and subjected to antimicrobial susceptibility tests by the disk diffusion method. Moreover, the D-test was performed to detect inducible clindamycin resistance. Data was analyzed using SPSS version 28. Binary logistic regression was used for the analysis, and *p*-values *≤* 0.05 with 95% CI were considered statistically significant. Antimicrobial susceptibility tests were analyzed using WHONET-2024 software.

**Results:**

In the current study, the overall prevalence of Gram-positive cocci was 32.0% (*n* = 96). The prevalence was significantly higher among PTB patients (60/150; 40.0%) compared with apparently healthy individuals (36/150; 24.0%). *Staphylococcus aureus* was the most common isolate (31.3%), followed by *Streptococcus pneumoniae* (29.2%) and coagulase-negative staphylococci (CONS) (16.7%) in both groups. Multidrug resistance (MDR) was observed in (84.2% and 81.8%) of *S. aureus* and 100% of CONS from PTB patients and apparently healthy individuals equally. Inducible clindamycin resistance (iMLSB) was detected among *S. aureus* (31.6% and 18.2%) and CONS (10.0% and 16.7%) in PTB patients and apparently healthy individuals, respectively. Overall methicillin resistance among GPC was 60.3%, with rates of 64.3% in PTB patients and 53.8% among apparently healthy individuals. A correlation analysis demonstrated a strong positive association between rifampicin-resistant *M. tuberculosis* and methicillin-resistant *S. aureus* (MRSA) (φ = 0.685). In multivariate analysis, age and educational status were independently associated with Gram-positive cocci (GPC) positivity among PTB patients. Patients aged ≥ 66 years had lower odds of GPC positivity (AOR: 0.27; 95% CI: 0.07–0.98), whereas participants with grades 5–8 education had higher odds (AOR: 8.65; 95% CI: 1.55–34.40). Cigarette smoking was inversely associated with GPC isolation (AOR: 0.43; 95% CI: 0.18–0.97). Among apparently healthy individuals, grades 5–8 education was more likely to harbor GPC (AOR: 7.75; 95% CI: 1.26–24.57).

**Conclusion:**

This study demonstrates a high burden of Gram-positive cocci colonization among PTB patients, frequent MDR and methicillin resistance, and inducible clindamycin resistance. The strong association between rifampicin-resistant Mtb among PTB patients and MRSA highlights the clinical relevance of coexisting drug resistance. The substantial burden of antimicrobial resistance underscores the need for routine D-tests, bacterial surveillance, and targeted infection control, especially among high-risk groups such as PTB patients.

**Supplementary Information:**

The online version contains supplementary material available at 10.1186/s12866-026-05071-y.

## Introduction

Tuberculosis (TB) remains a major public-health problem in Ethiopia, which continues to be listed among the World Health Organization’s high-burden countries for TB and TB/HIV [1, 2]. Concomitant bacterial infections and colonization of the respiratory tract by non-tuberculous pathogens are increasingly recognized among people with active PTB and can complicate clinical presentation, delay recovery, and increase morbidity and mortality. Gram-positive cocci- particularly *Staphylococcus aureus* and other *Staphylococci* and *Enterococci* are important causes of community- and healthcare-associated respiratory, skin/soft-tissue and invasive infections. The presence of these pathogens and others in PTB patients has implications for clinical management and infection control strategies [3, 4]. Further, infection with multiple pathogens elicits immune responses that differ from single-pathogen infections and may consequently alter disease severity and clinical outcomes [5].

Pulmonary tuberculosis patients frequently experience bacterial co-infections that can exacerbate disease progression, prolong treatment time, and worsen clinical outcomes, particularly in low-resource settings where diagnostic challenges and immune compromise are prevalent; such co-infections have been linked to higher morbidity and mortality, delayed recovery, and increased healthcare burden compared with PTB alone [4, 6]. Gram-positive cocci such as *S. aureus* and *Streptococcus* species are among the most commonly isolated bacteria from respiratory specimens and healthy carrier sites, showing significant carriage and disease potential in both clinical and community populations [7].

Antimicrobial resistance (AMR) among Gram-positive cocci compounds these clinical challenges. Methicillin-resistant *S. aureus* (MRSA), mediated most commonly by the mecA gene, limits beta-lactam therapeutic options and is associated with worse outcomes and greater healthcare costs. Equally important for clinical practice is inducible macrolide-lincosamide-streptogramin B (iMLSB) resistance- the phenotype in which isolates that appear erythromycin-resistant, but clindamycin-susceptible in routine testing can express resistance to clindamycin upon induction. The D-test is a simple phenotypic assay recommended for routine laboratories to detect inducible clindamycin resistance and thus to prevent clinical treatment failure when clindamycin is used empirically. Failure to identify iMLSB strains can lead to apparent clindamycin susceptibility in vitro but therapeutic failure in vivo [8, 9].

Hospitalization and repeated exposure to antibiotics, especially macrolides such as erythromycin-create strong selective pressure that favors the emergence and persistence of bacterial strains harboring *erm* resistance determinants. The e*rm* genes encode rRNA methyltransferases that methylate the 23 S rRNA, reducing binding of macrolides, lincosamides, and streptogramin B antibiotics and producing an MLSB resistance phenotype [10]. The *erm* methyltransferases catalyze mono- or demethylation of a specific adenine (A2058) in domain V of 23 S rRNA, sterically hindering antibiotic binding to the peptidyl transferase center [11]. These *erm* genes are among the most frequently detected mediators of MLSB resistance in clinical isolates, with inducible and constitutive expression patterns contributing to variable phenotypes [12].

In Ethiopia, recent studies document a substantial and variable prevalence of both MRSA and inducible clindamycin resistance among clinical staphylococcal isolates, highlighting the local relevance of these resistance mechanisms for empirical therapy and stewardship programs. Because clindamycin remains a valuable, affordable option for skin, soft tissue, and certain respiratory infections, accurate detection of iMLSB phenotypes is particularly important in resource-limited settings where alternative agents may be unavailable or costly. Similarly, detections of MRSA carriage or infection among PTB patients have implications for transmission control within clinics and hospitals that manage a large number of infectious TB cases [13, 14].

There remains a substantial evidence gap in Ethiopia regarding comparative data on the burdens of colonization/infection rates of Gram-positive cocci, and their methicillin and inducible clindamycin resistance patterns, as well as the factors contributing to their emergence between GeneXpert-positive PTB patients and apparently healthy individuals to explore their potential contribution to treatment outcomes in PTB patients relative to the control group. National antimicrobial resistance surveillance efforts are constrained by limited diagnostic capacity, shortages of skilled laboratory personnel, fragmented reporting systems, and persistent resource limitations [15, 16]. These challenges are further compounded by the prioritization of high-burden communicable diseases, inadequate policy-level commitment to AMR monitoring, and the widespread misuse of antibiotics in both community and clinical settings [17]. As a result, reliable data on resistance trends among clinically important Gram-positive cocci remain scarce among PTB patients and apparently healthy individuals. Therefore, this study aimed to determine the prevalence of Gram-positive cocci, methicillin resistance, and inducible clindamycin resistance among PTB patients and apparently healthy individuals in Northwest Ethiopia.

## Materials and methods

### Study area and setting

This study was conducted among pulmonary tuberculosis patients and apparently healthy individuals in three major referral and teaching hospitals and their respective community located in the Amhara National Regional State, Northwest Ethiopia. These are Debre Markos Comprehensive Specialized Hospital (DMCSH), University of Gondar Comprehensive Specialized Hospital (UoGCSH), and Felege Hiwot Comprehensive Specialized Hospital (FHCSH), and along with their respective surrounding communities. These hospitals are among the largest tertiary healthcare facilities in the region, serving as referral centers for millions of people residing in both urban and rural areas.

Debre Markos Comprehensive Specialized Hospital is situated in Debre Markos town, East Gojjam Zone, approximately 300 km northwest of Addis Ababa. The hospital offers specialized medical and surgical services, serving as a teaching hospital for Debre Markos University. University of Gondar Comprehensive Specialized Hospital is located in Gondar town about 727 km from Addis Ababa is one of the oldest and largest teaching hospital in Ethiopia. Felege Hiwot Comprehensive Specialized Hospital is found in Bahir Dar city, the capital of Amhara National Regional State, about 565 km northwest of Addis Ababa. It is a major referral and teaching hospital and provides comprehensive healthcare services to a large catchment population. All the three hospitals serve as referral centers for patients suspected of having tuberculosis and provide laboratory diagnostic services, including culture, GeneXpert MTB/RIF and antimicrobial susceptibility testing.

### Study design and period

A cross-sectional study was conducted to assess the prevalence of Gram-positive cocci (both colonization and infection) and their inducible clindamycin and methicillin susceptibility patterns among PTB patients and apparently healthy individuals from April 01, 2023, to May 30, 2025.

### Study population

The study population consisted of two groups: individuals diagnosed with tuberculosis and attending tuberculosis diagnosis and treatment centers at the selected comprehensive specialized hospitals, and apparently healthy individuals from the general community living in the same hospital catchment area.

### Sample size and sampling technique

There were 300 participants in all, 150 of whom tested positive for *M. tuberculosis* and another 150 apparently healthy individuals. Both study groups were equally chosen from Debre Markos, Bahir Dar, and Gondar (50 per site). The Mtb-positive participants were equally recruited from the three comprehensive specialized hospitals (50 per hospital) conveniently. Similarly, 50 apparently healthy participants were selected from each of the three study areas general community using a convenience sampling technique.

### Study variables

#### Dependent variables

- Prevalence of Gram-positive cocci (GPC)

#### Independent variables

##### Socio-demographic factors

- Age, sex, residence, educational status, occupation, economic status

##### Clinical and health-related factors

- TB status (PTB patient or apparently healthy individual) and TB patient contact history.
- HIV status and MTB rifampicin susceptibility status.
- Presence of comorbidity (e.g., diabetes, hypertension, pneumonia, asthma, urinary tract infection, malnutrition, hepatitis, chronic kidney disease, rheumatoid arthritis).
- Smoking habit, alcohol consumption, previous antibiotic use and source of drinking water.

### Eligibility criteria

#### Inclusion criteria

##### Pulmonary tuberculosis patients

Pulmonary tuberculosis patients eligible for inclusion were individuals newly diagnosed with pulmonary tuberculosis using the GeneXpert assay who were aged 8 years or older and attending comprehensive specialized hospitals in the Amhara National Regional State.

##### Apparently healthy individuals

Individuals eligible for inclusion were those with no clinical symptoms suggestive of tuberculosis, no known history of active TB, and no other comorbid conditions; who fulfilled the Ethiopian blood donors’ eligibility criteria; and who were aged between 18 and 65 years and living within the community in the same hospital catchment area.

#### Exclusion criteria

Patients with extrapulmonary tuberculosis or those already receiving anti-tuberculosis treatment were excluded from the study. In addition, critically ill patients who were unable to provide sputum samples and individuals who had received any antibiotics within two weeks prior to sample collection were not included.

### Operational definitions

Bacteriologically confirmed tuberculosis: Tuberculosis diagnosed in a biological specimen by a WHO-approved molecular test such as Xpert MTB/RIF [18].

Methicillin-resistant *S. aureus* (MRSA): *S aureus* with a zone of inhibition ≤ 21 mm for cefoxitin (FOX, 30 µg) on Mueller-Hinton Agar after 18–24 h of incubation [19].

Multidrug Resistance (MDR): Defined as Gram-positive cocci non-susceptibility to at least one antimicrobial agent in three or more antimicrobial classes, such as β-lactams, macrolides, fluoroquinolones, aminoglycosides, tetracyclines, lincosamides, glycopeptides, oxazolidinones, sulfonamides, nitrofurans, amphenicols in accordance with WHONET-2024 software criteria based on the international expert proposal by Magiorakos et al. [20, 21].

Inducible clindamycin resistance (iMLS_B_) phenotype: Gram-positive cocci isolates resistant to erythromycin and susceptible to clindamycin with a D-shaped zone of inhibition around the clindamycin disc [9, 19].

Constitutive clindamycin resistance (cMLS_B_) phenotype: Gram-positive cocci isolates were resistant to both drugs erythromycin (ERY) and clindamycin (CLI) [22].

Macrolide streptogramin (MS) phenotype: Gram-positive cocci showed resistance to erythromycin (ERY), but susceptible to clindamycin (CLI), with no evidence of a D-shaped zone of inhibition [23].

Sensitive (S) phenotype: Gram-positive cocci were sensitive to both ERY and CLI [19].

### Data collection and processing

#### Sociodemographic and clinical data collection

Socio-demographic and clinical data were collected using a structured and pre-tested questionnaire administered by trained healthcare personnel. Information included age, sex, residence, income, TB patient contact history, HIV status, alcohol drinking and smoking habit.

#### Specimen collection

Sputum was aseptically collected using sterile, leak-proof 50 ml Falcon tubes from both PTB patients and healthy individuals in accordance with standard operating procedures. Participants were instructed to expectorate deep cough sputum into sterile, leak-proof containers. The specimens were transported to the respective microbiology laboratories within one hour of collection for *M. tuberculosis* screening and rifampicin susceptibility profile.

#### Specimen processing

Sputum samples from tuberculosis-suspected participants were initially screened for *M. tuberculosis* and rifampicin resistance using the GeneXpert MTB/RIF assay [24]. An additional sputum specimen was collected from participants with confirmed *M. tuberculosis* infection and transported on ice to maintain bacterial viability for the isolation of Gram-positive cocci other than *M. tuberculosis*.

#### Isolation and identification of bacteria

Bacterial cultures were performed on blood agar and chocolate agar plates. Sputum specimens were inoculated onto each medium and incubated at 35–37 °C for 18–24 h, with chocolate agar maintained in a 5% CO₂-enriched atmosphere. Primary isolation and presumptive identification were based on colony morphology, hemolytic patterns on blood agar, and Gram staining. Mixed cultures suspected of contains more than one bacterium were sub-cultured to obtain pure colonies for subsequent identification.

The definitive identification of Gram-positive cocci was performed from pure cultures using standard biochemical tests [25]. Catalase test was performed to distinguish *Staphylococci* from *Streptococci*; slide coagulase test was performed to differentiate *S. aureus* from coagulase-negative staphylococci (CONS). Mannitol salt agar and the novobiocin susceptibility test were additionally employed for the characterization of staphylococcal isolates. Hemolysis on blood agar, along with pyrrolidinyl arylamidase (PYR) testing, bacitracin susceptibility, bile esculin hydrolysis, optochin sensitivity, bile solubility, and CAMP (Christie, Atkins, and Munch-Petersen) tests, were employed to accurately identify the *Streptococcus* species.

### Antimicrobial susceptibility test

Antimicrobial susceptibility test (AST) was performed for all isolates using the Kirby-Bauer disk diffusion method on Mueller-Hinton agar (MHA) in accordance with Clinical and Laboratory Standards Institute (CLSI) guidelines. For fastidious organisms such as *S. pneumoniae*, Mueller-Hinton agar supplemented with 5% defibrinated sheep blood was used [19]. From each pure isolate, 3–5 morphologically identical colonies were emulsified in 5 mL sterile normal saline and adjusted to a 0.5 McFarland turbidity standard. A sterile cotton swab was dipped into the suspension, and excess fluid was removed by rotating the swab against the inner wall of the tube. A uniform lawn inoculum was prepared on the surface of MHA. Antibiotic disks were applied to the plate surface, and plates were incubated at 37 °C for 18–24 h.

After incubation, the inhibition zone diameters (in millimeters) were measured using a calibrated ruler and interpreted in relation to the CLSI-2024 breakpoints. Results were reported as susceptible (S), intermediate (I), or resistant (R). Antibiotic panels were selected based on the likely pathogens, local availability, commonly prescribed agents in the study area, and CLSI-2024 recommendations [19, 26]. The following antimicrobials (Oxoid, LTD, UK) were tested for Gram-positive bacteria: Tetracycline (TCY, 30 µg), Erythromycin (ERY, 30 µg), Cefoxitin (FOX, 30 µg), Cefazolin (CZO, 30 µg), Cefuroxime (CXM, 30 µg), Cefepime (FEP), Penicillin (PEN, 10U), Ceftazidime (CAZ, 30 µg), Ciprofloxacin (CIP, 5 µg), Ceftriaxone (CRO, 30 µg), Clindamycin (CLI, 2 µg), Nitrofurantoin (NIT, 300 µg), Levofloxacin (LVX, 5 µg), Trimethoprim/sulfamethoxazole (SXT, 1.25/23.75 µg), Norfloxacin (NOR, 10 µg), Doxycycline (DOX, 30 µg), Chloramphenicol (CHL, 30 µg), Azithromycin (AZM, 15 µg), Linezolid (LNZ, 30 µg), Vancomycin (VAN, 30 µg) and Ceftaroline (CPT, 30 µg).

#### Detections of methicillin resistance among Gram-positive cocci (GPC)

The methicillin resistance pattern of Gram-positive cocci was confirmed phenotypically using cefoxitin disc (FOX, 30 µg). The plates were incubated aerobically at 35 °C for 18–24 h on MHA. The interpretations of the result were done for each coccus based on the CLSI-2024 guideline. *S. aureus* ATCC 29,213 and *S. pneumoniae* 49,619 were used as quality control strains [27].

#### D-test for inducible clindamycin resistance and interpretations

All isolates that were erythromycin resistant by the Kirby-Bauer disc diffusion method were subjected to D-test for inducible clindamycin resistance. It was performed using erythromycin (15 µg) and clindamycin (2 µg) discs that were positioned 15–20 mm apart from one another. Positive inducible clindamycin resistance (iMLSB phenotype) was defined as the appearance of a flattened clindamycin zone between clindamycin and erythromycin producing a D-shape with erythromycin resistance (D-test positive). For instance, *S. aureus* isolates showing circular zones of inhibition with a diameter of ≤ 13 mm for erythromycin (ERY) and ≥ 21 mm for clindamycin (CLI) without a D-shaped zone along ERY was interpreted as negative for inducible resistance (D-test negative) [28].

### Data quality assurance

All reagents, antibiotic discs, and culture media were inspected for appropriate storage conditions and expiration dates before use. A pre-test was conducted to ensure the completeness and consistency of the questionnaire. All laboratory procedures were performed in accordance with standard operating procedures. The quality of prepared culture media, biochemical tests, and antimicrobial susceptibility tests was verified using reference strains: *S. aureus* (ATCC 29213), *S. aureus* (ATCC 25923), and *S. pneumoniae* (ATCC 49619). The sterility and performance of each prepared culture medium were also evaluated before processing clinical specimens.

### Data analysis and interpretations

Data were entered into SPSS software, version 28 for analysis. Bivariate logistic regression was performed to identify factors associated with the presence of bacteria. Multivariate logistic regression was performed on variables with *p*-values *≤* 0.25 in the bivariate logistic regression. Multicollinearity between all covariates were assessed using the Variance Inflation Factor (VIF) prior to fitting the multivariable logistic regression model (VIF < 5). Finally, multivariate logistic regression analysis was performed to calculate the adjusted odds ratio (AOR) with 95% confidence intervals. A *p-*value of ≤ 0.05 with 95%CI was considered statistically significant. The Hosmer and Lemeshow goodness-of-fit test was used for model fitness. The Phi (Φ) coefficient was used to describe the strength and direction of association between *M. tuberculosis* and Gram-positive cocci. Antimicrobial susceptibility tests were analyzed using WHONET-2024 software.

## Results

### Sociodemographic characteristics of study participants

A total of 300 participants were enrolled in the study, comprising 150 GeneXpert-positive PTB patients and 150 apparently healthy individuals. Overall, 59.7% were male and 40.3% were female, with a higher proportion of males among healthy individuals (68.7%) than PTB patients (50.7%). Rifampicin resistance was detected in 19/150 (12.7%) of *M. tuberculosis* isolates. The majority of participants were aged 16–40 years (54.0%), while children (8–15 years) and older adults (*≥* 66 years) were observed only among PTB patients. More than half of the participants were living in urban areas (55.7%), although rural residence was more common among PTB patients (53.3%). Data on educational status showed that 26.4% had college-level education or above, while 23.3% were illiterate. The proportion of farmers was 31.3% and merchant accounted for 36.4%. Among PTB patients, 39 (26.0%) reported history of smoking habit, and alcohol consumption, 68 (45.3%). The data also showed that 52 (34.7%) of the PTB patients were HIV-positive. On the other hand, data on family size showed that a larger proportion of both groups lived in households with more than five family members, accounting for 90 (60.0%) of PTB patients and 108 (72.0%) of apparently healthy individuals. The majority of the participants in both groups had income in the range of 3,001–6,000 Ethiopian Birr (ETB) accounting for 77 (51.3%) PTB patients and 102 (68.0%) apparently healthy individuals (Table 1).

**Table 1:** Socio-demographic characteristics of pulmonary tuberculosis patients in selected comprehensive specialized hospitals and apparently healthy individuals in the community in Northwest Ethiopia (*N* = 300)

Categories of variables	Tuberculosis patients, *n* (%)	Apparently healthy individuals, *n* (%)	Total, *n* (%)
Sex			
Male	76 (50.7)	103 (68.7)	179 (59.7)
Female	74 (49.3)	47 (31.3)	121 (40.3)
Age group (in years)			
8–15	22 (14.7)	-	22 (7.3)
16–30	32 (21.3)	58 (38.7)	90 (30.0)
31–40	28 (18.7)	44 (29.3)	72 (24.0)
41–65	35 (23.3)	48 (32.0)	83 (27.7)
* ≥* 66	33 (22.0)	-	33 (11.0)
Residence			
Urban	70 (46.7)	97 (64.7)	167 (55.7)
Rural	80 (53.3)	53 (35.3)	133 (44.3)
Study area			
Gondar	50 (33.3)	50 (33.3)	100 (33.3)
Bahir Dar	50 (33.3)	50 (33.3)	100 (33.3)
Debre Markos	50 (33.3)	50 (33.3)	100 (33.3)
Educational status			
Illiterate	43 (28.7)	27 (18.0)	70 (23.3)
Grade 1–4	15 (10.0)	30 (20.0)	45 (15.0)
Grade 5–8	24 (16.0)	12 (8.0)	36 (12.0)
Grade 9–12	27 (18.0)	43 (28.7)	70 (23.3)
College & above	41 (27.3)	38 (25.3)	79 (26.4)
Occupation			
Government employed	33 (22.0)	43 (28.7)	76 (25.3)
Merchant	41 (27.3)	68 (45.3)	109 (36.4)
Farmer	61 (40.7)	33 (22.0)	94 (31.3)
Daily laborer	2 (1.3)	3 (2.0)	5 (1.7)
Student	4 (2.7)	3 (2.0)	7 (2.3)
Others	9 (6.0)	-	9 (3.0)
Family size			
< 5	60 (40.0)	42 (28.0)	102 (34.0)
* ≥* 5	90 (60.0)	108 (72.0)	198 (66.0)
Income (ETB)			
* ≤* 3,000	44 (29.3)	18 (12.0)	62 (20.7)
3,001–6,000	77 (51.3)	102 (68.0)	179 (59.7)
* ≥* 6,001	29 (19.3)	30 (20.0)	59 (19.6)
Total	150 (50.0)	150 (50.0)	300 (100)

### Prevalence of Gram-positive cocci

Overall, Gram-positive cocci were isolated from 60 PTB patients, giving a prevalence of (60/150; 40.0%; 95%CI: 32.2%- 47.8%), compared with 36 apparently healthy individuals, corresponding to a prevalence of (36/150; 24.0%; 95%CI: 17.2%- 30.8%). Gram-positive cocci were isolated from 26 (34.2%) males and 34 (46.0%) females from PTB patients. The highest prevalence was observed among children aged 8–15 years, where 13 of 22 participants were positive (60.0%), followed by those aged 16–30 years with 13 positives (40.6%) and those older than 66 years with 13 positives (39.4%). Gram-positive cocci were most frequently isolated among those with grade 5–8 education (15/24; 62.5%), followed by grade 1–4 (8/15; 53.3%), whereas the lowest prevalence was observed among college and above educated participants (13/41; 31.7%).

Among apparently healthy individuals, Gram-positive cocci were isolated from 25 males (24.3%) and 11 females (23.4%). The highest prevalence was observed in the 31-40-year age group, with 13 positives (29.6%), followed by those aged 41–65 years (13/48; 27.0%), whereas the lowest prevalence was observed among participants aged 16–30 years (10/58; 17.2%). Urban residents accounted for 20 positive isolates (20.6%), while 16 rural residents were positive (31.2%). With respect to educational status among apparently healthy individuals, the highest prevalence was observed among those with grade 5–8 education (8/12; 66.7%), while the lowest prevalence was noted among college and above (4/38; 10.5%) (Table 2).

**Table 2:** Prevalence of Gram-positive cocci among PTB patients in selected comprehensive specialized hospitals and apparently healthy individuals in the community in Northwest Ethiopia (*N* = 300)

Categories of variables	Gram-positive cocci among PTB patients (*n* = 150)			Gram-positive cocci among apparently healthy individuals (*n* = 150)		
	Positive, *n* (%)	Negative, *n* (%)	Total, *n* (%)	Positive, *n* (%)	Negative, *n* (%)	Total, *n* (%)
Sex						
Male	26 (34.2)	50 (65.8)	76 (50.7)	25 (24.3)	78 (75.7)	103 (69.7)
Female	34 (46.0)	40 (54.0)	74 (49.3)	11 (23.4)	36 (76.6)	47 (31.3)
Age group (in years)						
8–15	13 (60.0)	9 (40.0)	22 (14.7)	-	-	-
16–30	13 (40.6)	19 (59.4)	32 (21.3)	10 (17.2)	48 (82.8)	58 (38.7)
31–40	11 (39.3)	17 (60.7)	28 (18.7)	13 (29.6)	31 (70.4)	44 (29.3)
41–65	10 (28.6)	25 (71.4)	35 (23.3)	13 (27.0)	35 (73.0)	48 (32.0)
* ≥* 66	13 (39.4)	20 (60.6)	33 (22.0)	-	-	-
Residence						
Urban	29 (41.4)	41 (58.6)	70 (46.7)	20 (20.6)	77 (79.4)	97 (64.7)
Rural	31 (38.7)	49 (61.3)	80 (53.3)	16 (31.2)	37 (69.8)	53 (35.3)
Study area						
Gondar	19 (38.0)	31 (62.0)	50 (33.3)	12 (24.0)	38 (76.0)	50 (33.3)
Bahir Dar	21 (42.0)	29 (58.0)	50 (33.3)	13 (26.0)	37 (74.0)	50 (33.3)
Debre Markos	20 (40.0)	30 (60.0)	50 (33.3)	11 (22.0)	39 (78.0)	50 (33.3)
Educational status						
Illiterate	14 (32.6)	29 (67.4)	43 (28.7)	9 (33.3)	18 (66.7)	27 (18.0)
Grade 1–4	8 (53.3)	7 (46.7)	15 (10.0)	4 (13.3)	26 (86.7)	30 (20.0)
Grade 5–8	15 (62.5)	9 (37.5)	24 (16.0)	8 (66.7)	4 (33.3)	12 (8.0)
Grade 9–12	10 (37.0)	17 (63.0)	27 (18.0)	11 (25.6)	32 (74.4)	43 (28.7)
College & above	13 (31.7)	28 (68.3)	41 (27.3)	4 (10.5)	34 (89.5)	38 (25.3)
Occupation						
Government employed	9 (27.3)	24 (72.7)	33 (22.0)	5 (11.6)	38 (88.4)	43 (28.7)
Merchant	17 (41.5)	24 (59.5)	41 (27.3)	19 (28.0)	49 (72.0)	68 (45.3)
Farmer	23 (37.7)	38 (62.3)	61 (40.7)	10 (30.3)	23 (69.7)	33 (22.0)
Daily laborer	1 (5.0)	1 (50.0)	2 (1.3)	1 (33.3)	2 (66.7)	3 (2.0)
Student	3 (75.0)	1 (25.0)	4 (2.7)	1 (33.3)	2 (66.7)	3 (2.0)
Others	7 (77.8)	2 (22.2)	9 (6.0)	0 (0.0)	0 (0.0)	-
Family size						
< 5	27 (45.0)	33 (55.0)	60 (40.0)	6 (14.3)	36 (85.7)	42 (28.0)
* ≥* 5	33 (36.7)	57 (63.3)	90 (60.0)	30 (27.8)	78 (72.2)	108 (72.0)
Income (ETB)						
* ≤* 3,000	22 (50.0)	22 (50.0)	44 (29.3)	4 (22.2)	14 (77.8)	18 (12.0)
3,001–6,000	29 (37.7)	48 (62.3)	77 (51.3)	30 (29.4)	72 (70.6)	102 (68.0)
* ≥* 6,001	9 (31.0)	20 (69.0)	29 (19.3)	2 (6.7)	28 (93.3)	30 (20.0)
Total	60 (40.0)	90 (60.0)	150 (100)	36 (24.0)	114 (76.0)	150 (100)

*ETB* Ethiopian Birr, 1United States Dollar (USD)=155 ETB

### Type and magnitude of Gram-positive cocci

**Fig. 1:**
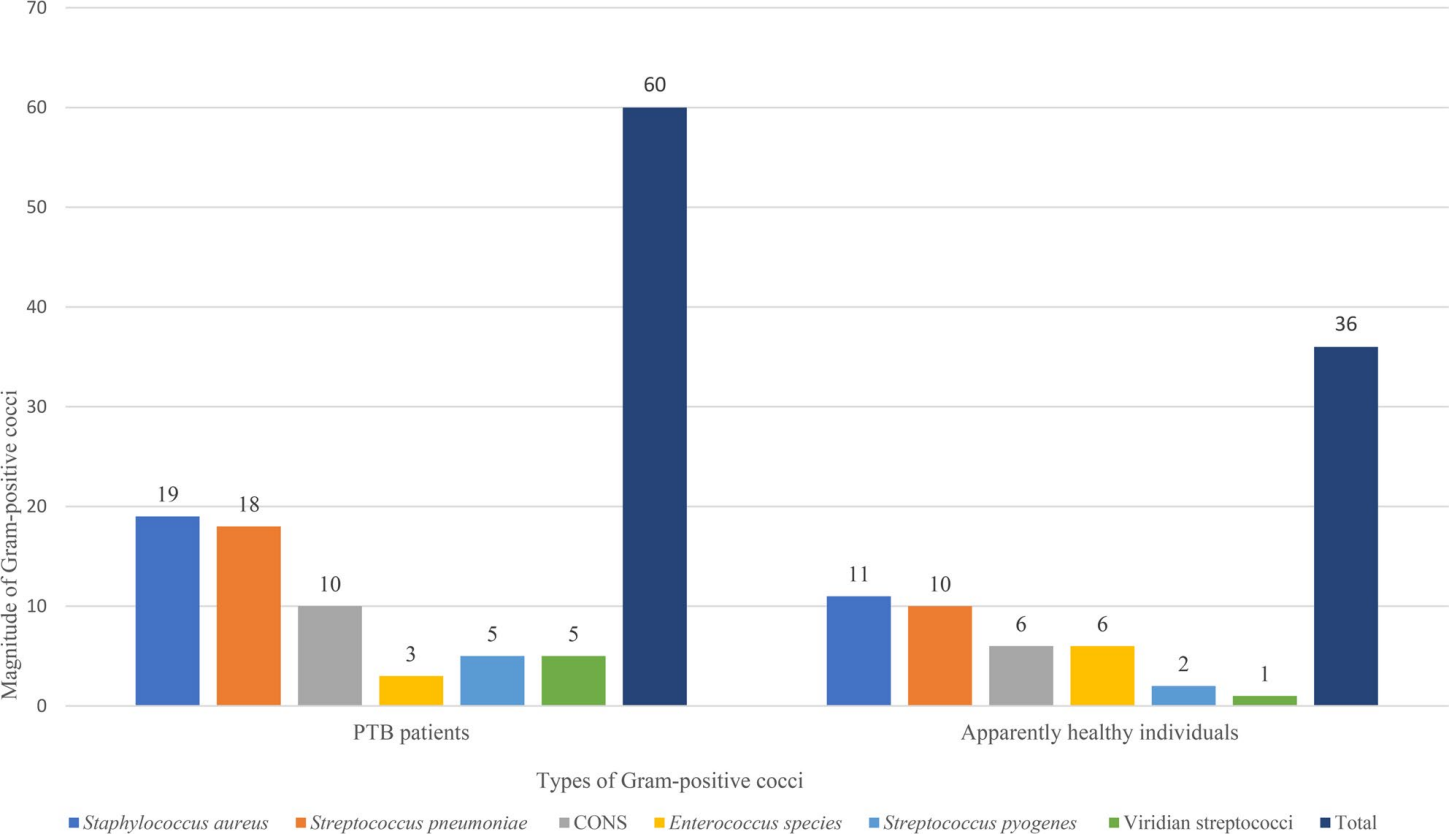
Type and magnitude of Gram-positive cocci among PTB patients in selected comprehensive specialized hospitals and apparently healthy individuals in the community in Northwest Ethiopia (*N* = 96)

A total of 96/300 (32.0%) Gram-positive cocci were isolated. Of these, 60 (40.0%) (95%CI: 32.2%- 47.8%) were recovered from PTB patients and 36 (24.0%) (95%CI: 17.2%- 30.8%) from apparently healthy individuals. *Staphylococcus aureus* was the most frequently isolated organism, accounting for 30 (31.3%) isolates, with comparable proportions among PTB patients (19/60, 31.7%) and healthy individuals (11/36, 30.5%). This was followed by *Streptococcus pneumoniae*, which comprised 28 isolates (29.2%), including 18 (30.0%) from PTB patients and 10 (27.8%) from healthy individuals. Coagulase-negative staphylococci (CONS) were identified in 16 cases (16.7%), equally distributed between PTB patients (10/60, 16.7%) and healthy individuals (6/36, 16.7%). *Enterococcus* species accounted for 9 isolates (9.4%), with a higher proportion among healthy individuals (6/36, 16.7%) than PTB patients (3/60, 5.0%). *Streptococcus pyogenes* and viridians streptococci were less common, contributing 7 (7.3%) and 6 (6.3%) isolates overall, respectively (Fig. 1).

### Antimicrobial susceptibility pattern of Gram-positive cocci

Antimicrobial resistance among Gram-positive cocci was widespread, particularly among isolates from PTB patients. *S. aureus* from PTB patients (*n* = 19) and apparently healthy individuals (*n* = 11) showed universal resistance to tetracycline, cefazolin, cefepime, ceftazidime, and ceftriaxone. High resistance was also observed to penicillin (89.5%). Methicillin resistance was common, observed in 57.9% of PTB patients and 36.4% of apparently healthy individuals. In contrast, resistance to nitrofurantoin, doxycycline, linezolid, and ceftaroline remained low (≤ 9.1%). *S. aureus* isolates were fully susceptible to ciprofloxacin. Coagulase-negative staphylococci from PTB patients showed complete resistance to cefazolin, cefuroxime, cefepime, ceftriaxone, and penicillin. Moreover, high resistance was observed to azithromycin (60.0%), erythromycin (50.0%), and tetracycline (50.0%). Susceptibility was best preserved for linezolid (80.0%), ciprofloxacin (80.0%), and doxycycline (70.0%). Coagulase-negative staphylococci from healthy individuals also demonstrated universal resistance to the tested cephalosporins and penicillin, but retained higher susceptibility to doxycycline, linezolid, and chloramphenicol (each 83.3%).

*Streptococci* and *Enterococci* displayed heterogeneous but clinically relevant resistance patterns, with preserved activity of last-line agents. *S. pneumoniae* isolates from PTB patients (*n* = 18) showed high resistance to chloramphenicol (88.9%) and tetracycline (66.7%), but complete susceptibility to levofloxacin, linezolid, and vancomycin. Isolates from healthy individuals (*n* = 10) remained fully susceptible to these agents. *S. pyogenes* from PTB patients (*n* = 5) was fully susceptible to penicillin, tetracycline, levofloxacin, vancomycin, and linezolid, but showed resistance to cefotaxime, cefoxitin, and clindamycin (3/5 each). Isolates from healthy individuals (*n* = 2) were largely susceptible, with resistance limited to erythromycin, cefoxitin, and clindamycin (1/2 each). Among *Enterococcus* species from PTB patients (*n* = 3) exhibited complete resistance to ampicillin and high resistance to tetracycline, erythromycin, and ciprofloxacin (2/3 each), yet remained fully susceptible to vancomycin and linezolid. Viridians streptococci from PTB patients (*n* = 5) showed resistance mainly to cefepime, tetracycline, and erythromycin (3/5 each), while all isolates retained susceptibility to vancomycin and linezolid (Tables 3 and 4).

**Table 3:** Antimicrobial susceptibility pattern of Gram-positive cocci among PTB patients in selected comprehensive specialized hospitals in Northwest Ethiopia (*N* = 60)

GPC		TCY	ERY	FOX	CZO	CXM	FEP	KAZ	CIP	CRO	CLI
*S. aureus* *n* = 19	R	19(100)	11(57.9)	11(57.9)	19(100)	11(57.9)	19(100)	19(100)	0(0.0)	19(100)	7(36.8)
	I	0(0.0)	3(15.8)	0(0.0)	0(0.0)	0(0.0)	0(0.0)	0(0.0)	6(31.6)	0(0.0)	3(15.8)
	S	0(0.0)	5(26.3)	8(42.1)	0(0.0)	8(42.1)	0(0.0)	0(0.0)	13(68.4)	0(0.0)	9(47.4)
CONS, *n* = 10	R	5(50.0)	5(50.0)	9(90.0)	10(100)	10(100)	10(100)	NT	1(10.0)	10(100)	4(40.0)
	I	2(20.0)	2(20.0)	0(0.0)	0(0.0)	0(0.0)		NT	1(10.0)	0(0.0)	1(10.0)
	S	3(30.0)	3(30.0)	1(10.0)	0(0.0)	0(0.0)	0(0.0)	NT	8(80.0)	0(0.0)	5(50.0)

		**TCY**	**ERY**	**FOX**	**CTX**	**VAN**	**FEP**	**AMP**	**CIP**	**DOX**	**CLI**
*S. pneumonia*, *n* = 18	R	12(66.7)	4(22.5)	NT	NT	0(0.0)	NT	NT	NT	5(27.8)	2(11.1)
	I	3(16.7)	10(55.6)	NT	NT	2(11.1)	NT	NT	NT	7(38.9)	6(33.3)
	S	3(16.7)	4(22.5)	NT	NT	16(88.9)	NT	NT	NT	6(33.3)	10(55.6)
*S. pyogenes*, *n* = 5	R	0(0.0)	2(40.0)	3(60.0)	3(60.0)	0(0.0)	0(0.0)	2(40.0)	NT	1(20.0)	3(60.0)
	I	0(0.0)	0(0.0)	0(0.0)	0(0.0)	0(0.0)	0(0.0)	0(0.0)	NT	2(40.0)	0(0.0)
	S	5(100)	3(60.0)	2(40.0)	2(40.0)	5(100)	5(100)	2(40.0)	NT	2(40.0)	2(40.0)
*Enterococcus* species, *n* = 3	R	2(66.7)	2(66.7)	1(33.3)	NT	0(0.0)	NT	3(100)	2(66.7)	NT	1(33.3)
	I	0(0.0)	1(33.3)	0(0.0)	NT	0(0.0)	NT	0(0.0)	0(0.0)	NT	0(0.0)
	S	1(33.3)	0(0.0)	2(66.7)	NT	3(100)	NT	0(0.0)	1(33.3)	NT	2(66.7)
Viridians streptococci, *n* = 5	R	3(60.0)	3(60.0)	2(40.0)	2(40.0)	0(0.0)	3(60.0)	NT	NT	0(0.0)	2(40.0)
	I	1(20.0)	0(0.0)	0(0.0)	1(20.0)	0(0.0)	0(0.0)	NT	NT	2(40.0)	0(0.0)
	S	1(20.0)	2(40.0)	3(60.0)	2(40.0)	5(100)	2(40.0)	NT	NT	3(60.0)	3(60.0)

**GPC**		**NIF**	**SXT**	**NOR**	**CHL**	**DOX**	**AZM**	**LNZ**	**CPT**	**PEN**	
*S. aureus, n*=19	R	1(5.3)	5(26.3)	0(0.0)	2(10.5)	1(5.3)	10(52.6)	1(5.3)	1(5.3)	17(89.5)	
	I	0(0.0)	7(36.8)	10(52.6)	9(47.4)	9(47.4)	7(36.8)	1(5.3)	6(31.6)	0(0.0)	
	S	18(94.7)	7(36.8)	9(47.4)	8(42.1)	9(47.4)	2(10.5)	17(89.5)	12(63.2)	2(10.5)	
CONS, *n*=10	R	1(10.0)	1(10.0)	0(0.0)	4(40.0)	0(0.0)	6(10.0)	0(0.0)	NT	10(100)	
	I	1(10.0)	4(40.0)	7(70.0)	2(20.0)	3(30.0)	2(20.0)	2(20)	NT	0(0.0)	
	S	8(80.0)	5(50.0)	3(30.0)	4(40.0)	7(70.0)	2(20.0)	8(80.0)	NT	0(0.0)	

		**NIF**	**SXT**	**NOR**	**CHL**	**LVX**	**AZM**	**LNZ**	**CPT**	**PEN**	
*S. pneumonia, n*=18	R	NT	5(27.8)	NT	16(88.9)	0(0.0)	NT	0(0.0)	0(0.0)	NT	
	I	NT	7(38.9)	NT	0(0.0)	0(0.0)	NT	4(22.5)	2(11.1)	NT	
	S	NT	6(33.3)	NT	2(11.1)	18(100)	NT	14(77.8)	16(88.9)	NT	
*S. pyogenes, n*=5	R	NT	NT	NT	1(20.0)	0(0.0)	0(0.0)	0(0.0)	NT	0(0.0)	
	I	NT	NT	NT	2(40.0)	0(0.0)	0(0.0)	0(0.0)	NT	0(0.0)	
	S	NT	NT	NT	2(40.0)	5(100)	5(100)	5(100)	NT	5(100)	
*Enterococcus *species, *n*=3	R	0(0.0)	NT	0(0.0)	0(0.0)	NT	NT	0(0.0)	NT	0(0.0)	
	I	0(0.0)	NT	1(33.3)	0(0.0)	NT	NT	0(0.0)	NT	0(0.0)	
	S	3(100)	NT	2(66.7)	3(100)	NT	NT	3(100)	NT	3(100)	
Viridians streptococci, *n*=5	R	NT	NT	NT	0(0.0)	0(0.0)	0(0.0)	0(0.0)	NT	NT	
	I	NT	NT	NT	0(0.0)	0(0.0)	0(0.0)	0(0.0)	NT	NT	
	S	NT	NT	NT	5(100)	5(100)	5(100)	5(100)	NT	NT	

*GPC* Gram-positive cocci, *TCY* Tetracycline, *ERY* Erythromycin, *FOX* Cefoxitin, *CZO* Cefazolin, *CXM* Cefuroxime, *FEP* Cefepime, *PEN* Penicillin, *CAZ*  Ceftazidime, *KAN* Kanamycin, *CIP* Ciprofloxacin, *CRO* Ceftriaxone, *CLI*  Clindamycin, *NIT* Nitrofurantoin, *SXT* Trimethoprim/sulfamethoxazole, *GEN* Gentamycin, *NOR* Norfloxacin, *CHL* Chloramphenicol, *AZM *Azithromycin, *LNZ* Linezolid, *CPT* Ceftaroline,  *DOX* Doxycycline, *VAN* Vancomycin, *NT* Not tested

**Table 4:** Antimicrobial susceptibility pattern of Gram-positive cocci among apparently healthy individuals in the community in Northwest Ethiopia (*N* = 36)

GPC		TCY	ERY	FOX	CZO	CXM	FEP	KAZ	CIP	CRO	CLI
*S. aureus*, *n* = 11	R	11(100)	5(45.5)	4(36.4)	11(100)	5(45.5)	11(100)	11(100)	0(0.0)	11(100)	3(27.3)
	I	0(0.0)	2(18.2)	0(0.0)	0(0.0)	0(0.0)	0(0.0)	0(0.0)	0(0.0)	0(0.0)	2(18.2)
	S	0(0.0)	4(36.4)	7(63.6)	0(0.0)	6(54.5)	0(0.0)	0(0.0)	11(100)	0(0.0)	6(54.5)
CONS, *n* = 6	R	2(33.3)	3(50.0)	6(100)	6(100)	6(100)	6(100)	NT	1(16.7)	6(100)	2(33.3)
	I	2(33.3)	2(33.3)	0(0.0)	0(0.0)	0(0.0)	0(0.0)	NT	1(16.7)	0(0.0)	0(0.0)
	S	2(33.3)	1(16.7)	0(0.0)	0(0.0)	0(0.0)	0(0.0)	NT	4(66.7)	0(0.0)	4(66.7)

		**TCY**	**ERY**	**FOX**	**CTX**	**VAN**	**FEP**	**AMP**	**CIP**	**DOX**	**CLI**
*S. pneumoniae*, *n* = 10	R	6(60.0)	0(0.0)	NT	NT	0(0.0)	NT	NT	NT	4(40.0)	3(30.0)
	I	1(10.0)	5(50.0)	NT	NT	0(0.0)	NT	NT	NT	3(30.0)	2(20.0)
	S	3(30.0)	5(50.0)	NT	NT	10(100)	NT	NT	NT	3(30.0)	5(50.0)
*S. pyogenes*, *n* = 2	R	0(0.0)	1(50.0)	1(50.0)	0(0.0)	0(0.0)	0(0.0)	0(0.0)	NT	0(0.0)	1(50.0)
	I	1(50.0)	0(0.0)	0(0.0)	0(0.0)	0(0.0)	0(0.0)	0(0.0)	NT	0(0.0)	1(50.0)
	S	1(50.0)	1(50.0)	1(50.0)	2(100)	2(100)	2(100)	2(100)	NT	2(100)	0(0.0)
*Enterococcus* species, *n* = 6	R	3(50.0)	3(50.0)	2(33.3)	NT	1(16.7)	NT	3(50.0)	1(16.7)	NT	3(50.0)
	I	0(0.0)	1(16.7)	1(16.7)	NT	0(0.0)	NT	0(0.0)	0(0.0)	NT	0(0.0)
	S	2(33.3)	1(16.7)	3(50.0)	NT	5(83.3)	NT	1(16.7)	5(83.3)	NT	3(50.0)
Viridians streptococci, *n* = 1	R	1(100)	1(100)	1(100)	0(0.0)	0(0.0)	0(0.0)	NT	NT	0(0.0)	1(100)
	I	0(0.0)	0(0.0)	0(0.0)	0(0.0)	0(0.0)	0(0.0)	NT	NT	0(0.0)	0(0.0)
	S	0(0.0)	0(0.0)	0(0.0)	1(100)	1(100)	1(100)	NT	NT	1(100)	0(0.0)

**GPC**		**NIF**	**SXT**	**NOR**	**CHL**	**DOX**	**AZM**	**LNZ**	**CPT**	**PEN**	
*S. aureus, *n=11	R	0(0.0)	0(0.0)	1(9.1)	1(9.1)	0(0.0)	5(45.5)	0(0.0)	1(9.1)	11(100)	
	I	0(0.0)	1(9.1)	1(9.1)	2(18.2)	3(27.3)	3(27.3)	4(36.4)	3(27.3)	0(0.0)	
	S	11(100)	10(90.9)	9(81.8)	8(72.7)	8(72.7)	3(27.3)	7(63.6)	7(63.6)	0(0.0)	
CONS, n=6	R	0(0.0)	2(33.3)	0(0.0)	1(16.7)	0(0.0)	2(33.3)	0(0.0)	NT	6(100)	
	I	0(0.0)	1(16.7)	2(33.3)	0(0.0)	1(16.7)	1(16.7)	1(16.7)	NT	0(0.0)	
	S	6(100)	3(50.0)	4(66.7)	5(83.3)	5(83.3)	3(50.0)	5(83.3)	NT	0(0.0)	

		**NIF**	**SXT**	**NOR**	**CHL**	**LVX**	**AZM**	**LNZ**	**CPT**	**PEN**	
*S. pneumonia, *n=10	R	NT	1(10.0)	NT	4(40.0)	0(0.0)	NT	0(0.0)	0(0.0)	NT	
	I	NT	2(20.0)	NT	2(20.0)	0(0.0)	NT	0(0.0)	2(20.0)	NT	
	S	NT	7(70.0)	NT	4(40.0)	10(100)	NT	10(100)	8(80.0)	NT	
*S. pyogenes, *n=2	R	NT	NT	NT	0(0.0)	0(0.0)	0(0.0)	0(0.0)	NT	0(0.0)	
	I	NT	NT	NT	0(0.0)	1(50.0)	0(0.0)	0(0.0)	NT	0(0.0)	
	S	NT	NT	NT	2(100)	1(50.0)	2(100)	2(100)	NT	2(100)	
*Enterococcus *species, n=6	R	1(16.7)	NT	1(16.7)	1(16.7)	NT	NT	0(0.0)	NT	1(16.7)	
	I	0(0.0)	NT	0(0.0)	0(0.0)	NT	NT	1(16.7)	NT	0(0.0)	
	S	5(83.3)	NT	5(83.3)	5(83.3)	NT	NT	5(83.3)	NT	5(83.3)	
Viridians streptococci, n=1	R	NT	NT	NT	1(100)	0(0.0)	0(0.0)	0(0.0)	NT	NT	
	I	NT	NT	NT	0(0.0)	0(0.0)	0(0.0)	0(0.0)	NT	NT	
	S	NT	NT	NT	0(0.0)	1(100)	1(100)	1(100)	NT	NT	

*S. pyogenes, *n=2

*GPC* Gram-positive cocci, *TCY* Tetracycline, *ERY* Erythromycin, *FOX* Cefoxitin, *CZO* Cefazolin, *CXM* Cefuroxime, *FEP* Cefepime, *PEN* Penicillin, *CAZ*  Ceftazidime, *KAN* Kanamycin, *CIP* Ciprofloxacin, *CRO* Ceftriaxone, *CLI*  Clindamycin, *NIT* Nitrofurantoin, *SXT* Trimethoprim/sulfamethoxazole, *GEN* Gentamycin, *NOR* Norfloxacin, *CHL* Chloramphenicol, *AZM* Azithromycin, *LNZ* Linezolid, *CPT* Ceftaroline,  *DOX* Doxycycline, *VAN* Vancomycin, *NT* Not tested

### Multidrug resistance (MDR) pattern of Gram-positive cocci

Among the Gram-positive cocci isolated in this study (*n* = 96), a high burden of multidrug resistance was observed particularly among staphylococcal species. *S. aureus* exhibited MDR in 16/19 (84.2%) of isolates from PTB patients and 9/11 (81.8%) from apparently healthy individuals. Similarly, CONS showed 100% MDR in isolates from both PTB patients and healthy individuals. In contrast, no MDR was identified among *S. pneumoniae*,* S. pyogenes*, and viridians streptococci in either group. *Enterococcus* species demonstrated MDR in 66.7% of isolates from PTB patients and 16.7% from healthy individuals (Table 5).

**Table 5:** Multidrug resistance (MDR) profile of Gram-positive cocci among PTB patients in selected comprehensive specialized hospitals and apparently healthy individuals in the community in Northwest Ethiopia (*n* = 96)

Types of Gram-positive cocci	Number of drug classes tested	MDR profiles of isolates among PTB patients (*n* = 60)		MDR profiles of isolates among apparently healthy individuals (*n* = 36)	
		Number of isolates tested	MDR, *n* (%)	Number of isolates tested	MDR, *n* (%)
*S. aureus*	15	19	16 (84.2)	11	9 (81.8)
Coagulase- negative staphylococci	15	10	10 (100)	6	6 (100)
*S. pneumoniae*	9	18	0 (0.0)	10	0 (0.0)
*S. pyogenes*	11	5	0 (0.0)	2	0 (0.0)
*Enterococcus* species	10	3	2 (66.7)	6	1 (16.7)
Viridians streptococci	11	5	0 (0.0)	1	0 (0.0)

*MDR* Multidrug resistance

### Inducible clindamycin resistance among Gram-positive cocci (GPC)

Among the *S. aureus* isolates tested (*n* = 30) for MLSB resistance phenotypes, 19 were recovered from PTB patients and 11 from apparently healthy individuals. In PTB patients, the most frequent phenotype was constitutive MLSB resistance (cMLSB), observed in 7 isolates (36.8%), followed by inducible MLSB resistance (iMLSB) in 6 isolates (31.6%). The susceptible (S) phenotype (erythromycin- and clindamycin-sensitive) accounted for 4 isolates (21.0%), while the macrolide streptogramin **(**MS) phenotype was least common, detected in 2 isolates (10.5%). Among apparently healthy individuals, the susceptible phenotype predominated, accounting for 4 isolates (36.4%), followed by cMLSB resistance in 3 isolates (27.3%). Both iMLSB and MS phenotype were each observed in 2 isolates (18.2%). Overall, cMLSB resistance was the most common MLSB phenotype (33.3%), followed by iMLSB and fully susceptible phenotypes, each accounting for 26.7% of isolates, whereas the MS phenotype was the least frequent (13.3%).

**Fig. 2:**
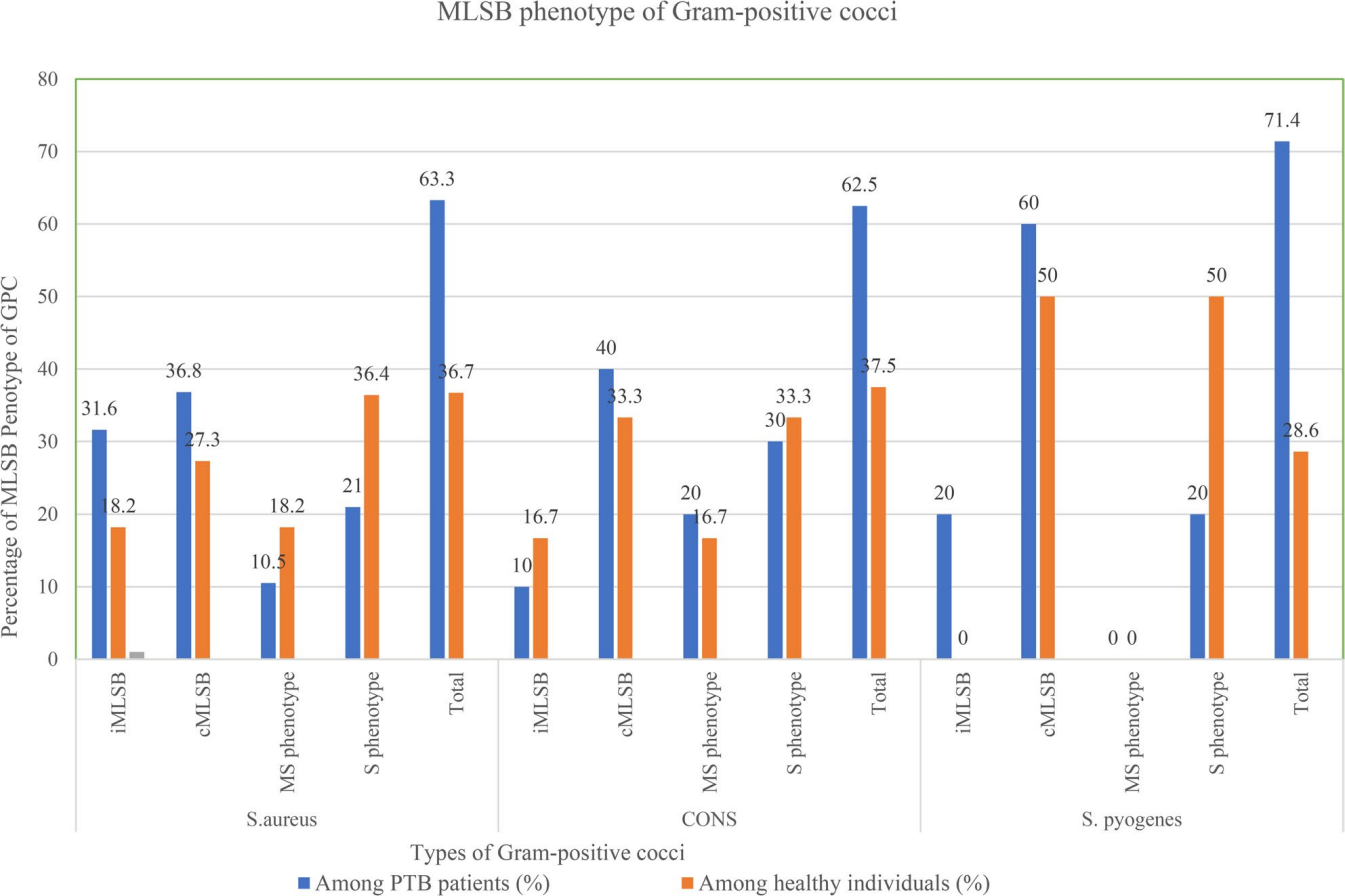
Macrolide-lincosamide-streptogramin B (MLSB) resistance of Gram-positive cocci among PTB patients in selected comprehensive specialized hospitals and apparently healthy individuals in the community in Northwest Ethiopia (*N* = 52); iMLSB: ERY−R, CLN− S (D test positive); cMLSB: ERY−R, CLN− R; MS phenotype: ERY−R, CLN− S (D test negative); S phenotype: ERY−S, CLN−S

Among CONS and *S. pyogenes*, cMLSB resistance remained the most prevalent phenotype, observed in 37.5% and 57.1% of isolates, respectively. Inducible clindamycin resistance (iMLSB) was detected in 12.5% of CONS and 14.3% of *S. pyogenes*, occurring predominantly among PTB patients. The MS phenotype was absent in *S. pyogenes*, while susceptible (S) phenotypes were more frequent among isolates from apparently healthy individuals (Fig. 2).

### Methicillin susceptibility patten among Gram-positive cocci (GPC)

**Fig. 3:**
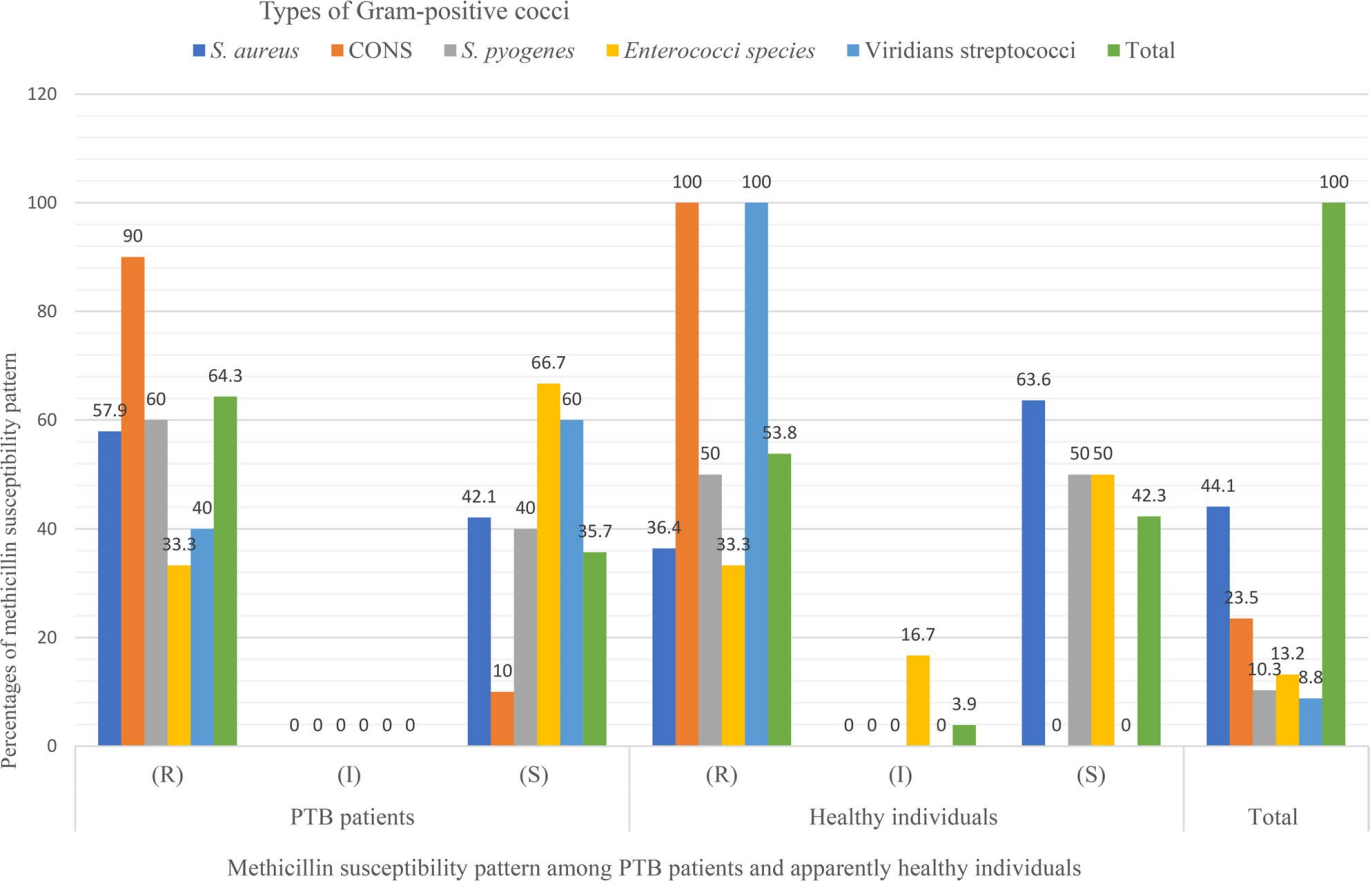
Methicillin susceptibility pattern of Gram-positive cocci among PTB patients in selected comprehensive specialized hospitals and apparently healthy individuals in the community in Northwest Ethiopia (*N* = 68), R: Resistant; I: Intermediate; S: sensitive

Among the 68 Gram-positive cocci isolates tested for methicillin susceptibility, a high proportion of methicillin resistance was observed 41/68(60.3%). Of the 42 GPC isolates from PTB patients, 27 (64.3%) were methicillin-resistant, while 15 (35.7%) were sensitive. In comparison, among the 26 isolates from apparently healthy individuals, 14 (53.8%) were resistant, 11 (42.3%) were sensitive, and only one (3.9%) showed intermediate susceptibility. Coagulase-negative staphylococci showed 90% and 100% methicillin resistance in PTB patients and healthy individuals, respectively. Methicillin-resistant *S. aureus* (MRSA) was more frequent among PTB patients (57.9%) than among healthy individuals (36.4%). Resistance was also observed among *S. pyogenes* isolates from both groups (60.0% in PTB patients and 50.0% in healthy individuals). *Enterococci* species demonstrated comparatively lower resistance, with one-third of isolates resistant in both groups, while viridians streptococci showed moderate resistance among PTB patients (40.0%) (Fig. 3).

### Correlation of drug resistance between *M. tuberculosis* and Gram-positive cocci

The correlation analysis between *M. tuberculosis* (Mtb) rifampicin susceptibility and methicillin or inducible clindamycin resistance in Gram-positive cocci among PTB patients revealed variable associations. Methicillin-resistant *S. aureus* (MRSA) showed a strong positive correlation with rifampicin resistance (φ = 0.685), with 90% of MRSA isolates occurring in rifampicin-resistant TB cases. In contrast, methicillin resistance in coagulase-negative staphylococci had a weak correlation (φ = 0.167). Inducible clindamycin resistance in *S. aureus* showed a weak positive correlation (φ = 0.121), while in CONS it was stronger (φ = 0.667), indicating most resistant isolates were associated with rifampicin-resistant *M. tuberculosis*. *S. pyogenes* inducible clindamycin resistance exhibited a strong negative correlation (φ= -0.667), and vancomycin resistance in *S. pneumoniae* showed a weak negative correlation (φ= -0.158) (Table 6).

**Table 6:** Correlation between drug susceptibility test of *M. tuberculosis* and methicillin, inducible clindamycin resistance pattern of Gram-positive cocci among PTB patients in selected comprehensive specialized hospitals in Northwest Ethiopia

Categories		Mtb rifampicin susceptibility pattern		Total, n (%)	Phi (φ) correlation coefficient
		Resistant, n (%)	Sensitive, n (%)		
Methicillin susceptibility of *S. aureus*	Resistance	9 (90.0)	1 (10.0)	10 (52.6)	0.685
	Sensitive	2 (22.2)	7 (77.8)	9 (47.4)	
Methicillin susceptibility of CONS	Resistance	2 (22.2)	7 (77.8)	9 (90.0)	0.167
	Sensitive	0 (0.0)	1 (100)	1 (10.0)	
Inducible clindamycin susceptibility pattern of *S. aureus*	Resistance	4 (66.7)	2 (33.3)	6 (31.6)	0.121
	Sensitive	7 (53.8)	6 (46.2)	13 (68.4)	
Inducible clindamycin susceptibility pattern of CONS	Resistance	1 (100)	0 (0.0)	1 (10.0)	0.667
	Sensitive	1 (11.1)	8 (88.9)	9 (90.0)	
Inducible clindamycin susceptibility pattern of *S. pyogenes*	Resistance	1 (33.3)	2 (66.7)	3 (60.0)	-0.667
	Sensitive	2 (100)	0 (0.0)	2 (40.0)	
Vancomycin susceptibility pattern of *S. pneumoniae*	Resistance	0 (0.0)	2 (100)	2 (11.1)	-0.158
	Sensitive	3 (18.8)	13 (81.2)	16 (88.9)	

*CONS* Coagulase−negative staphylococci, *Phi* (φ) correlation coefficient measures the strength and direction of association between two binary variables. φ ranges from −1 to +1: values close to +1 indicate a strong positive association, values close to −1 indicate a strong negative association, and values near 0 indicate little or no association

### Factors associated with respiratory Gram-positive cocci infections and/or colonization

In the bivariate analysis, age group and educational status were significantly associated with GPC positivity among PTB patients. Individuals aged 41–65 years were less likely to be GPC-positive than those aged 8–15 years (COR: 0.28; 95% CI: 0.09–0.85; *p* = 0.025), whereas participants with grades 5–8 education had higher odds of GPC positivity compared with those with college education and above (COR: 3.59; 95% CI: 1.24–10.31; *p* = 0.018) (Table 7). Cigarette smoking showed a significant inverse association with GPC isolation (COR: 0.42; 95% CI: 0.18–0.94; *p* = 0.036) (Supplementary Table 1). Among apparently healthy individuals, educational status and occupation were significantly associated with GPC carriage: illiterate participants (COR: 4.25; 95% CI: 1.15–15.73; *p* = 0.030) and those with grades 5–8 education (COR: 17.00; 95%CI: 3.35–33.10; *p* = 0.001) had higher odds of GPC positivity compared with those with college education and above, and farmers were more likely to be GPC-positive than government employees (COR: 3.30; 95%CI: 1.01–10.88; *p* = 0.049) (Supplementary Table 2).

**Table 7:** Bivariate and multivariate analysis of sociodemographic factors for Gram-positive cocci among PTB patients in selected comprehensive specialized hospitals in Northwest Ethiopia (*N* = 150)

Variables	Gram-positive cocci among PTB patients		COR (95%CI)	*P*-value	AOR (95%CI)	*P*-value
	Positive, *n* (%)	Negative, *n* (%)				
Sex						
Male	26 (34.2)	50 (65.8)	0.61 (0.32–1.20)	0.144	0.62 (0.29–1.35)	0.236
Female	34 (46.0)	40 (54.0)	1			
Age group (in years)						
8–15	13 (60.0)	9 (40.0)	1			
16–30	13 (40.6)	19 (59.4)	0.47 (0.16–1.43)	0.185	0.37 (0.11–1.33)	0.130
31–40	11 (39.3)	17 (60.7)	0.45 (0.14–1.40)	0.167	0.44 (0.12–1.67)	0.229
41–65	10 (28.6)	25 (71.4)	0.28 (0.09–0.85)	0.025	0.26 (0.06–1.08)	0.064
* ≥* 66	13 (39.4)	20 (60.6)	0.45 (0.15–1.35)	0.155	0.27 (0.07–0.98)	0.047
Residence						
Urban	29 (41.4)	41 (58.6)	1			
Rural	31 (38.7)	49 (61.3)	0.89 (0.46–1.72)	0.738		
Study area						
Gondar	19 (38.0)	31 (62.0)	0.92 (0.41–2.04)	0.839		
Bahir Dar	21 (42.0)	29 (58.0)	1.08 (0.49–2.42)	0.832		
Debre Markos	20 (40.0)	30 (60.0)	1			
Educational status						
Illiterate	14 (32.6)	29 (67.4)	1.04 (0.44–2.45)	0.933	1.12 (0.22–5.66)	0.895
Grade 1–4	8 (53.3)	7 (46.7)	2.46 (0.71–8.55)	0.144	3.75 (0.70-19.94)	0.121
Grade 5–8	15 (62.5)	9 (37.5)	3.59 (1.24–10.31)	0.018	8.65 (1.55–34.40)	0.014
Grade 9–12	10 (37.0)	17 (63.0)	1.27 (0.44–3.67)	0.650	1.67 (0.46–6.05)	0.429
College & above	13 (31.7)	28 (68.3)	1		1	
Occupation						
Government employed	9 (27.3)	24 (72.7)	1		1	
Merchant	17 (41.5)	24 (59.5)	1.90 (0.71–5.07)	0.206	0.53 (0.12–2.36)	0.411
Farmer	23 (37.7)	38	1.61 (0.64–4.03)	0.310	1.28 (0.27–6.02)	0.755
Daily laborer	1 (5.0)	1 (50.0)	2.67 (0.13–24.50)	0.504	0.34 (0.01–9.10)	0.521
Student	3 (75.0)	1 (25.0)	8.00 (0.73–37.70)	0.088	1.02 (0.06–18.01)	0.987
Others͌	7 (77.8)	2 (22.2)	9.33 (1.38–22.90)	0.012	8.85 (1.53–31.32)	0.015
Family size						
< 5	27 (45.0)	33 (55.0)	1			
* ≥* 5	33 (36.7)	57 (63.3)	0.71 (0.37–1.38)	0.308		
Income (ETB)						
* ≤* 3,000	22 (50.0)	22 (50.0)	2.22 (0.83–5.94)	0.112	0.31 (0.04–2.06)	0.225
3,001–6,000	29 (37.7)	48 (62.3)	1.34 (0.54–3.33)	0.527	0.87 (0.14–5.29)	0.886
* ≥* 6,001	9 (31.0)	20 (69.0)	1		1	
Total	60 (40.0)	90 (60.0)				

*ETB* Ethiopian Birr; 1 USD= 155 ETB, *COR* Crude odds ratio, *AOR * Adjusted odds ratio, *CI* Confidence interval, *Others* pension, elderly

In the multivariate model, age, educational status, and occupation remained independently associated with GPC positivity among PTB patients. Patients aged ≥ 66 years were less likely to be GPC-positive than those aged 8–15 years (AOR: 0.27; 95%CI: 0.07–0.98; *p* = 0.047), while those with grades 5–8 education had markedly higher odds of GPC positivity compared with participants with college education and above (AOR: 8.65; 95% CI: 1.55–34.40; *p* = 0.014) (Table 7). Cigarette smoking also remained inversely associated with GPC isolation (AOR: 0.43; 95%CI: 0.18–0.97; *p* = 0.046) (Supplementary Table 1). Among apparently healthy individuals, age group, and educational status remained significant predictors: participants aged 16–30 years had lower odds of GPC positivity than those aged 41–65 years (AOR: 0.29; 95%CI: 0.09–0.87; *p* = 0.028), and individuals with grades 5–8 education were significantly more likely to harbor GPC than those with college education and above (AOR: 7.75; 95%CI: 1.26–24.57; *p* = 0.027) (Supplementary Table 2).

## Discussion

In the present study, Gram-positive cocci (GPC) were isolated among 40.0% (60/150) (95%CI: 32.2%- 47.8%) of pulmonary tuberculosis (PTB) patients compared with 24.0% (36/150) (95% CI: 17.2%- 30.8%) of apparently healthy individuals, indicating higher prevalence among those with active PTB. Within the PTB cohort, females showed somewhat higher isolation rate (46.0%) than males (34.2%), and the highest prevalence was observed in children aged 8–15 years (60.0%) and young adults aged 16–30 years (40.6%). Moreover, lower educational status and lower income correlated with higher GPC prevalence, suggesting the socioeconomic and demographic influences on colonization or infection. Among the apparently healthy individuals, males and females had almost similar prevalence (24%), and the highest prevalence occurred in the 31-40-years age group (29.6%), with rural residents showing higher rates (31.2%) than urban residents (20.6%). These patterns reflect a substantial carriage or infection burden of Gram-positive cocci in both clinical tuberculosis and community settings.

Among Gram-positive cocci, *S. aureus* was the most frequently isolated organism (31.3%), with comparable proportions among PTB patients (31.7%) and healthy individuals (30.5%), followed by coagulase-negative staphylococci (CONS) (16.7%), which were equally distributed between the two groups. Similar findings have been reported in Ethiopia, where GPC constitute important clinical isolates; for instance, a study from Arsho Advanced Medical Laboratory in Addis Ababa GPC identified in 12.6% of 792 clinical specimens, predominantly *S. aureus* (54%) and CONS (42%). The overall prevalence was lower than in the present PTB cohort, likely due to differences in study populations and sample types [29]. Systematic reviews and population-based studies further highlighted the substantial burden of GPC and antimicrobial resistance in Ethiopia and East Africa, with *S. aureus* carriage commonly documented in both hospital and community settings [30–33]. In addition, previous studies have shown that sputum from PTB patients frequently yields bacteria other than *M. tuberculosis*, including both Gram-positive and Gram-negative organisms. These findings suggest that PTB patients and healthy controls may harbor additional bacterial flora, which may represent colonization or secondary infection and may influence clinical outcomes and antimicrobial use [3, 34–37].

Multidrug resistance (MDR) in this study was predominantly observed among staphylococcal species. Both *S. aureus* and CONS showed very high MDR rates in isolates from PTB patients and apparently healthy individuals. In contrast, *S. pneumoniae*, *S. pyogenes*, and viridians streptococci exhibited no MDR in either group, while *Enterococcus* species showed moderate MDR, particularly among PTB patients. These findings are consistent with Ethiopian reports documenting endemic MDR among GPC, especially *Staphylococci*, where pooled analyses have shown MDR rates exceeding 60–70% for *S. aureus* and 64–78% for CONS [38, 39]. The high MDR rates observed here for *S. aureus* (84.2% in PTB patients; 81.8% in healthy individuals) and CONS (100% in both groups) align with reports from the Amhara region and other settings [14, 40].

Globally, *S. aureus*, particularly MRSA is recognized as a leading MDR pathogen in both healthcare and community settings, and together with *Enterococcus* species constitutes one of the most clinically significant Gram-positive threats to effective therapy [41, 42]. The widespread emergence of resistance to multiple antibiotic classes mirrors global trends and is exacerbated in settings with less regulated antibiotic use, such as in Ethiopia [43]. Although *Enterococcus* species show increasing MDR and occasional vancomycin resistance worldwide, their resistance burden generally remains lower than that of *Staphylococci* species [44]. In contrast, *S. pneumoniae* and other *Streptococci* are more often characterized by specific resistance profiles rather than broad MDR, influenced by vaccine implementation and different antibiotic selection pressures [42]. The marked predominance of MDR among *Staphylococci* species indicates their persistent role as the principal Gram-positive resistance challenge in Ethiopia.

In the present study, constitutive MLSB (cMLSB) resistance was the predominant phenotype among *S. aureus*, CONS, and *S. pyogenes*; however, inducible clindamycin resistance (iMLSB) constituted a substantial proportion of isolates, particularly among PTB patients. Overall, iMLSB was detected in 26.7% of *S. aureus* isolates (31.6% in PTB patients vs. 18.2% in apparently healthy individuals), with lower but notable rates in CONS (12.5%) and *S. pyogenes* (14.3%), mainly among PTB patients. Susceptible (S) phenotypes were more frequent in healthy individuals, and the macrolide streptogramin **(**MS) phenotype was least common, indicating that clinically relevant inducible resistance may be missed without routine D-testing. Comparable studies report wide geographic variability in iMLSB prevalence, including an African systematic review showing a 19.8% rate in *S. aureus* (2.9–44.0%), often higher in MRSA, and reports from India and Japan ranging from 10.7% to 31.9%, reflecting differences in antibiotic pressure, prescribing practices, and the distribution of *erm* genes such as *ermA* and *ermC* [45–47].

Consistent with previous reports, cMLSB resistance commonly dominates among clinical staphylococcal isolates, with studies from Iran and Turkey documenting frequencies of 92.8% and 63.9%, respectively, and much lower iMLSB and MS proportions [48, 49]. Although cMLSB was also the most frequent phenotype in the current study (33.3%), the relatively balanced distribution between cMLSB and iMLSB contrasts with the marked dominance reported elsewhere, likely reflecting differences in patient populations and local antimicrobial exposure; the observed iMLSB prevalence is comparable to findings from Debre Markos and University of Gondar hospitals (26%) [13, 50]. Among CONS and *S. pyogenes*, cMLSB also predominated, though the higher MLSB proportion in *S. pyogenes* differs from reports where the MS phenotype is more common, suggesting geographic or population-specific selective pressures [51–54]. These findings emphasize the need for routine D-testing and ongoing surveillance to guide appropriate clindamycin use and prevent therapeutic failure due to unrecognized inducible resistance [45, 49, 55, 56].

The high overall prevalence of methicillin resistance among Gram-positive cocci (60.3%) observed in this study is consistent with previous clinical and surveillance reports from Ethiopia. Substantial MRSA rates have been documented in Debre Markos town (36.9%) [50], and a national meta-analysis of CONS and MRSA isolates reported frequently high methicillin resistance, in some settings exceeding 60% [57]. Moreover, MRSA prevalence in *S. aureus* varies from lower community levels (17–28%) to markedly higher rates in hospital environments (> 50%) [58, 59], supporting the elevated proportions observed among PTB patients. Collectively, these findings indicate that the high drug resistance GPC burden among both PTB patients and apparently healthy individuals, reflects broader regional trends likely driven by antibiotic exposure and healthcare-related selective pressures.

Among the GPC isolates, MRSA showed a strong positive correlation with rifampicin-resistant *M. tuberculosis* (φ = 0.685), with 90% of MRSA isolates originating from rifampicin-resistant TB cases, whereas methicillin resistance in CONS showed only a weak association (φ = 0.167). Inducible clindamycin resistance demonstrated a weak positive correlation in *S. aureus* (φ = 0.121), but a stronger association in CONS (φ = 0.667), while *S. pyogenes* exhibited a strong negative correlation (φ= -0.667) and vancomycin resistance in *S. pneumoniae* showed a weak negative correlation (φ= -0.158). These patterns suggest a notable link between rifampicin-resistant Mtb and MRSA, with other Gram-positive resistance profiles showing weaker or inconsistent associations, reflecting heterogeneous bacterial ecology and antibiotic exposure in PTB patients. The possible reasons for a strong correlation between MRSA and rifampicin-resistant TB could be selection pressure, hospital-associated transmission dynamics, multidrug resistance co-selection, and shared risk factors such as prior antibiotic exposure, and immunocompromise with TB, which predispose to colonization and resistant organisms [60, 61]. Studies conducted in hospital settings, particularly in tuberculosis wards, have reported a markedly higher frequency of rifampicin-resistant MRSA isolates among PTB patients compared with those in other hospital wards. This observation suggests that sustained rifampicin exposure in these settings may exert selective pressure that promotes the emergence and persistence of this antimicrobial resistance phenotype [62].

In the current study, multivariate analysis revealed that sociodemographic factors, age and education level-were independently associated with colonization or infection by GPC among GeneXpert-positive PTB patients, with patients aged ≥ 66 years significantly less likely to be GPC-positive compared with those aged 8–15 years (AOR 0.27; *p* = 0.047) and individuals with only grades 5–8 education markedly more likely to harbor GPC than those with college education or above (AOR 8.65; *p* = 0.014). Cigarette smoking was inversely associated with GPC isolation (AOR 0.43; *p* = 0.046), and among apparently healthy individuals, younger age (16–30 years) was associated with lower odds of GPC positivity compared with ages 41–65 (*p* = 0.028) while mid-school education again conferred higher risk versus tertiary education (*p* = 0.027). These patterns align with Ethiopian and other studies reporting sociodemographic influences on Gram-positive bacteria carriage among illiterate participants had significantly higher odds of *S. aureus* infection, underscoring how lower education may be linked to higher bacterial carriage due to factors such as limited health literacy and hygiene practices [50, 63]. In contrast, a study from Niger found that age and other demographic variables were not significantly associated with GPC isolates, indicating heterogeneity across settings in socio-demographic predictors of carriage [64]. Other regional respiratory infection studies reported that age and behavioral factors (e.g., alcohol use) as significant predictors for culture-positive bacterial infections, while sociodemographic predictors such as education and occupation show variable associations across settings [65, 66]. Moreover, a systematic review across Africa found that GPC colonization and antibiotic resistance varied substantially with population and associated factors such as healthcare exposure, prior antibiotic use, and comorbidities, highlighting the complex interplay of demographic, behavioral and clinical determinants in GPC colonization and resistance [67].

Cigarette smoking showed an inverse association with GPC isolation in PTB patients in the current study. In contrast, broader evidence showing that smoking is a consistent associated factor for bacterial respiratory infections. Multiple studies and systematic reviews indicated that smoking compromises respiratory defenses, increasing susceptibility to bacterial pathogens like *S. pneumoniae* and other respiratory bacteria with up to 2 to 3-fold higher risk of respiratory tract infections in smokers versus non-smokers [68, 69]. Similarly, a meta-analysis of community-acquired pneumonia found smokers were at elevated risk for bacterial respiratory infection, and links smoking with increased nasopharyngeal carriage [70]. Socioeconomic factors like education and income are less consistently associated with the current finding of bacterial carriage contrasting with other studies [71, 72], possibly due to differences in exposure patterns, local transmission dynamics, and specific Gram-positive organisms studied, as well as methodological and population differences between studies.

The observed differences in GPC diversity, antimicrobial resistance patterns, and inducible clindamycin resistance between tuberculosis patients and apparently healthy individuals likely reflect variations in antimicrobial exposure and selective pressure. Healthy individuals mainly represent community carriage with limited antibiotic exposure, resulting in lower resistance selection, whereas PTB patients experience prolonged or repeated antimicrobial therapy that promotes the emergence and persistence of resistant strains. These findings highlight the need to strengthen antimicrobial stewardship and infection prevention, particularly among PTB patients.

### Limitations of the study

This study, focused on culturable bacterial species, potentially underestimates the true diversity of the respiratory microbiota. True sputum is difficult to obtain from healthy people, and the low microbial load in healthy lungs may lead to false-negative results. The healthy group was recruited using the blood donor screening checklist and therefore included only adults, whereas the PTB group comprised both children and older adults. Consequently, the groups were not age-matched, which may affect the comparability of bacterial carriage and resistance patterns. Thus, the findings should be interpreted with caution.

## Conclusion and recommendation

This study demonstrated a substantial burden of Gram-positive cocci (GPC) among pulmonary tuberculosis (PTB) patients and apparently healthy individuals, with a markedly higher prevalence among TB patients (40.0%) compared with healthy controls (24.0%). *S. aureus* and *S. pneumoniae* were the predominant pathogens in both groups, indicating their major role in respiratory colonization and infection. A high burden of multidrug resistance (MDR) was observed, especially among *Staphylococci*, with alarming rates of MDR in both PTB patients and healthy individuals. Methicillin resistance was highly prevalent among PTB patients, and inducible clindamycin resistance (iMLSB) was common, highlighting the importance of inclusions of routine D-test. The study further identified important epidemiological links between PTB and antimicrobial resistance, as MRSA and inducible clindamycin-resistant CONS showed strong positive correlations with rifampicin-resistant *M. tuberculosis*. These findings indicate that age and educational level-play a key role in GPC carriage, underscoring the need for targeted preventive strategies and antimicrobial resistance surveillance focused on high-risk groups.

The high prevalence of methicillin resistance, multidrug resistance, and inducible clindamycin resistance, particularly among PTB patients, emphasizes the need for strengthened antimicrobial stewardship and routine bacteriological surveillance in PTB care settings. Culture-based identification of GPC with comprehensive antimicrobial susceptibility testing, including D-test screening and methicillin resistance, should be integrated into routine clinical practice to guide targeted therapy and prevent inappropriate antibiotic use. Further molecular and genomic studies are warranted to clarify microbiome dynamics and resistance gene reservoirs influencing transmission and treatment outcomes.

## Supplementary Information

Supplementary Material 1.

Supplementary Material 2.

## Data Availability

All relevant data are within the manuscript and its supporting information files.

## Abbreviations

AMR: Antimicrobial Resistance
ATCC: American Type Culture Collection
CLSI: Clinical and Laboratory Standards Institute
CONS: Coagulase-negative staphylococci
DMCSH: Debre Markos Comprehensive Specialized Hospital
FHCSH: Felege-Hiwot Comprehensive Specialized Hospital
GPC: Gram-positive cocci
MDR: Multidrug Resistance
MRSA: Methicillin Resistant S. aureus
PTB: Pulmonary tuberculosis
UoGCSH: University of Gondar Comprehensive Specialized Hospital
