# Supplementary material for: Methicillin and inducible clindamycin resistance in Gram-positive cocci among GeneXpert-positive pulmonary tuberculosis patients and apparently healthy individuals in Northwest Ethiopia
Source: BMC Microbiol. 2026 Apr 18;26:517. doi: 10.1186/s12866-026-05071-y (PMC13224584; doi:10.1186/s12866-026-05071-y)
Supplement: Supplementary file 1 — Supplementary Material 1. [file 12866_2026_5071_MOESM1_ESM.docx]

| **Variables** | **Gram-positive cocci among PTB patients** | | **COR (95%CI)** | **P-value** | **AOR (95%CI)** | **P-value** |
| --- | --- | --- | --- | --- | --- | --- |
|  | **Positive, n (%)** | **Negative, n (%)** |  |  |  |  |
| **Underlying disease** |  |  |  |  |  |  |
| HIV/AIDS | 16 (30.8) | 36 (69.2) | 1.55 (0.29-8.33) | 0.606 | 1.56 (0.28-8.60) | 0.605 |
| Diabetes | 9 (50.0) | 9 (50.0) | 3.50 (0.56-21.66) | 0.178 | 3.69 (0.57-23.88) | 0.170 |
| UTI | 10 (55.6) | 8 (44.4) | 4.37 (0.70-27.16) | 0.113 | 4.72 (0.73-30.50) | 0.102 |
| Asthma | 8 (42.0) | 11 (58.0) | 2.54 (0.41-15.65) | 0.313 | 2.96 (0.46-19.08) | 0.254 |
| Pneumonia | 3 (30.0) | 7 (70.0) | 1.50 (0.18-11.92) | 0.702 | 2.02 (0.23-17.22) | 0.519 |
| Hypertension | 8 (50.0) | 8 (50.0) | 3.30 (0.54-22.30) | 0.185 | 3.62 (0.54-23.99) | 0.182 |
| Others* | 4 (50.0) | 4 (50.0) | 3.50 (0.43-28.44) | 0.241 | 4.65 (0.52-42.15) | 0.172 |
| No underlying disease | 2 (22.2) | 7 (77.8) | 1 |  | 1 |  |
| **Previous antibiotics use** |  |  |  |  |  |  |
| Yes | 32 (41.6) | 45 (58.4) | 0.87 (0.45-1.68) | 0.689 |  |  |
| No | 28 (38.4) | 45 (61.6) | 1 |  | 1 |  |
| **Cigarette smoking** |  |  |  |  |  |  |
| Yes | 10 (25.6) | 29 (74.4) | 0.42 (0.18-0.94) | 0.036 | 0.43 (0.18-0.97) | 0.046 |
| No | 50 (45.0) | 61 (55.0) | 1 |  | 1 |  |
| **Alcohol drinking** |  |  |  |  |  |  |
| Yes | 25 (36.8) | 43 (63.2) | 0.78 (0.40-1.51) | 0.462 |  |  |
| No | 35 (42.7) | 47 (57.3) | 1 |  |  |  |
| **Rifampicin susceptibility profile** |  |  |  |  |  |  |
| Resistant (R) | 4 (21.0) | 15 (79.0) | 3.12 (0.62-15.7) | 0.168 | 0.37 (0.07-1.91) | 0.237 |
| Sensitive (S) | 51 (42.5) | 69 (57.5) | 1.13 (0.33-3.89) | 0.850 | 1.02 (0.29-3.58) | 0.974 |
| Indeterminate (ID) | 5 (45.5) | 6 (54.5) | 1 |  | 1 |  |
| **Contact history with TB patients** |  |  |  |  |  |  |
| Yes | 11 (27.5) | 29 (72.5) | 2.12 (0.96-4.66) | 0.062 |  |  |
| No | 49 (44.5) | 61 (54.5) | 1 |  |  |  |
| **HIV status** |  |  |  |  |  |  |
| Positive | 16 (30.8) | 36 (69.2) | 1.83 (0.90-3.73) | 0.095 | 1.89 (0.92-3.89) | 0.083 |
| Negative | 44 (44.9) | 54 (55.1) | 1 |  | 1 |  |
| **Total** | **60 (40.0)** | **90 (60.0)** |  |  |  |  |

**Supplementary Table 1:** Bivariate and multivariate analysis of clinical and associated factors for Gram-positive cocci among PTB patients in selected comprehensive specialized hospitals in Northwest Ethiopia (N=150)

^COR: Crude odds ratio; AOR: Adjusted odds ratio; CI: Confidence interval; Others: Malnutrition, hepatitis, chronic kidney disease, rheumatoid arthritis^
