# Supplementary material for: Methicillin and inducible clindamycin resistance in Gram-positive cocci among GeneXpert-positive pulmonary tuberculosis patients and apparently healthy individuals in Northwest Ethiopia
Source: BMC Microbiol. 2026 Apr 18;26:517. doi: 10.1186/s12866-026-05071-y (PMC13224584; doi:10.1186/s12866-026-05071-y)
Supplement: Supplementary file 2 — Supplementary Material 2. [file 12866_2026_5071_MOESM2_ESM.docx]

**Supplementary Table 2**: Logistic regression analysis of associated factors for culturable Gram-positive cocci among apparently healthy individuals in the community in Northwest Ethiopia (N=150)

| **Categories of variables** | **Gram-positive cocci** **among apparently healthy individuals (n=150)** | | **COR (95%CI)** | **P-value** | **AOR (95%CI)** | **P-value** |
| --- | --- | --- | --- | --- | --- | --- |
|  | **Positive, n (%)** | **Negative, n (%)** |  |  |  |  |
| **Sex** |  |  |  |  |  |  |
| Male | 25 (24.3) | 78 (75.7) | 1 |  |  |  |
| Female | 11 (23.4) | 36 (76.6) | 0.95 (0.42-2.15) | 0.908 |  |  |
| **Age group (in years)** |  |  |  |  |  |  |
| 16-30 | 10 (17.2) | 48 (82.8) | 0.56 (0.22-1.42) | 0.224 | 0.29 (0.09-0.87) | 0.028 |
| 31-40 | 13 (29.6) | 31 (70.4) | 1.13 (0.45-2.80) | 0.793 | 0.78 (0.27-2.27) | 0.653 |
| 41-65 | 13 (27.0) | 35 (73.0) | 1 |  | 1 |  |
| **Residence** |  |  |  |  |  |  |
| Urban | 20 (20.6) | 77 (79.4) | 1 |  | 1 |  |
| Rural | 16 (31.2) | 37 (69.8) | 0.60 (0.28-1.29) | 0.192 | 1.03 (0.38-2.77) | 0.956 |
| **Study area** |  |  |  |  |  |  |
| Bahir Dar | 13 (26.0) | 37 (74.0) | 1.25 (0.49-3.13) | 0.640 |  |  |
| Gondar | 12 (24.0) | 38 (76.0) | 1.12 (0.44-2.84) | 0.812 |  |  |
| Debre Markos | 11 (22.0) | 39 (78.0) | 1 |  |  |  |
| **Educational status** |  |  |  |  |  |  |
| Illiterate | 9 (33.3) | 18 (66.7) | 4.25 (1.15-15.73) | 0.030 | 1.98 (0.38-10.20) | 0.413 |
| Grade 1-4 | 4 (13.3) | 26 (86.7) | 1.30 (0.29-5.72) | 0.722 | 0.55 (0.09-3.15) | 0.500 |
| Grade 5-8 | 8 (66.7) | 4 (33.3) | 17.00 (3.35-33.10) | 0.001 | 7.75 (1.26-24.57) | 0.027 |
| Grade 9-12 | 11 (25.6) | 32 (74.4) | 2.92 (0.84-10.12) | 0.091 | 1.23 (0.28-5.45) | 0.782 |
| College & above | 4 (10.5) | 34 (89.5) | 1 |  | 1 |  |
| **Occupation** |  |  |  |  |  |  |
| Government employed | 5 (11.6) | 38 (88.4) | 1 |  | 1 |  |
| Merchant | 19 (28.0) | 49 (72.0) | 2.94 (1.01-8.61) | 0.048 | 1.42 (0.23-8.86) | 0.704 |
| Farmer | 10 (30.3) | 23 (69.7) | 3.30 (1.01-10.88) | 0.049 | 1.92 (0.18-10.12) | 0.587 |
| Daily laborer | 1 (33.3) | 2 (66.7) | 3.80 (0.29-22.90) | 0.310 | 0.12 (0.01-3.42) | 0.215 |
| Student | 1 (33.3) | 2 (66.7) | 3.80 (0.29-22.90) | 0.310 | 0.53 (0.01-8.43) | 0.724 |
| **Source of drinking water** |  |  |  |  |  |  |
| Tap water | 20 (20.6) | 77 (79.4) | 1 |  |  |  |
| River and others | 16 (31.2) | 37 (69.8) | 1.66 (0.77-3.58) | 0.292 |  |  |
| **Family size** |  |  |  |  |  |  |
| < 5 | 6 (14.3) | 36 (75.7) | 1 |  |  |  |
| > 5 | 30 (27.8) | 78 (72.2) | 0.43 (0.17-1.13) | 0.880 |  |  |
| **Income (ETB)** |  |  |  |  |  |  |
| < 3,000 | 4 (22.2) | 14 (77.8) | 4.00 (0.65-24.55) | 0.134 | 0.36 (0.35-15.94) | 0.334 |
| 3,001-6,000 | 30 (29.4) | 72 (70.6) | 5.83 (1.30-26.05) | 0.021 | 6.72 (1.05-25.97) | 0.044 |
| > 6,001 | 2 (6.7) | 28 (93.3) | 1 |  | 1 |  |
| **Total** | **36 (24.0)** | **114 (76.0)** |  |  |  |  |

^COR: Crude odds ratio; AOR: Adjusted odds ratio; CI: Confidence interval; ETB: Ethiopian Birr; 1USD= 155 ETB^
